# A rare case of gastric metastasis originating from primary lung adenocarcinoma: a case report

**DOI:** 10.2144/fsoa-2022-0083

**Published:** 2023-06-01

**Authors:** Amal Khsiba, Manel Moalla, Narjes Abid, Amel Douggaz, Moufida Mahmoudi, Asma Ben Mohamed, Manel Yakoubi, Mouna Medhioub, Lamine Hamzaoui, Emna Chelbi, Mohamed Moussadek Azzouz

**Affiliations:** 1 Gastroenterology Department, Mohamed Taher Maamouri Hospital, Nabeul, Tunisia; 2 Pulmonology Department, Mohamed Taher Maamouri Hospital, Nabeul, Tunisia; 3 Pathology Department, Mohamed Taher Maamouri Hospital, Nabeul, Tunisia

**Keywords:** gastric metastasis, immunochemistry, lung cancer

## Abstract

Unlike liver and lung, the stomach is rarely a metastatic location for cancers. We report a case of a 62-year-old man known to have lung adenocarcinoma poorly differentiated presented with melena 1 month after diagnosis. Upper endoscopy revealed an ulcerated tumor in the prepyloric antrum. The diagnosis of gastric metastasis from pulmonary cancer was confirmed by the immunohistochemical staining for the thyroid transcriptional factor-1 and the pattern cytokeratine CK7+/CK20-. In conclusion, gastric metastasis from primary lung cancer is a rare phenomenon that every clinician must keep in mind.

## Plain language summary

A 62-year-old man known to have lung adenocarcinoma poorly differentiated presented with melena 1 month after diagnosis. Upper endoscopy revealed an ulcerated tumor in the prepyloric antrum. The diagnosis of gastric metastasis from pulmonary cancer was confirmed by histology.

Unlike liver and lung, the stomach is rarely a metastatic location for cancers. Cases of pulmonary cancer and breast cancer with gastric metastasis have been described in the literature. Usually, gastric metastasis is asymptomatic which explains the high rates of postmortem diagnosis. It is difficult to distinguish primary gastric cancer and gastric metastasis on clinical, endoscopic or radiological features. We hereby report a case of gastric metastasis with pulmonary primary cancer.

Aims: to highlight the importance of the patient's history and the role of immunochemistry to confirm diagnosis.

## Case report

**Figure 1.**
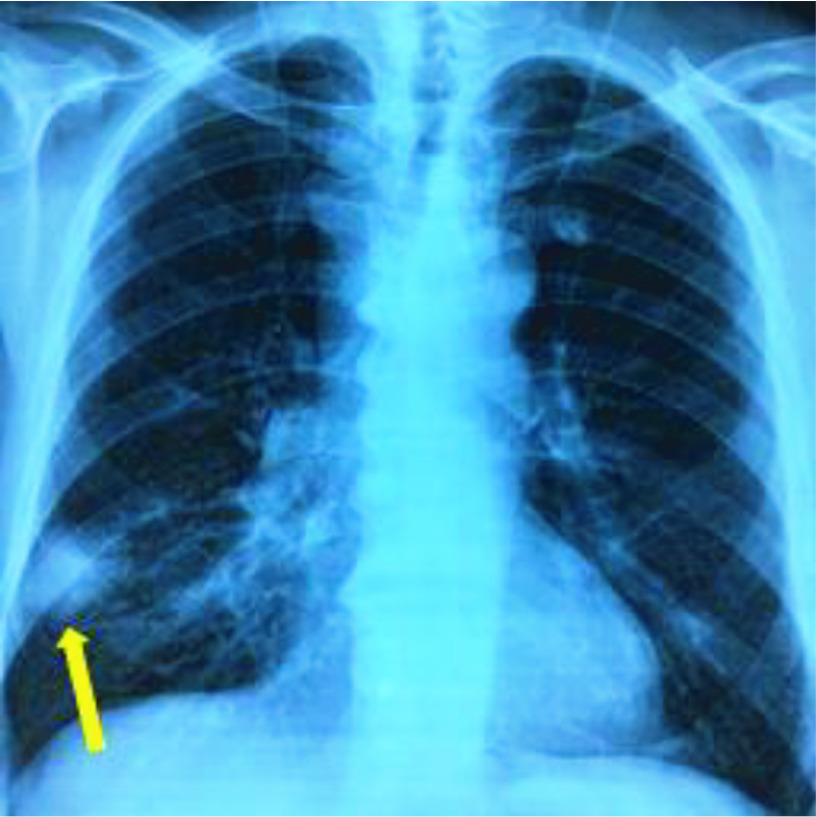
Chest x-ray showing a spiculated opacity in the right lung.

A 62-year-old male patient with a 50 year smoking history of about 50 packages of cigarettes per year, who underwent a duodenopancreatectomy for an ampulloma in 2010, was diagnosed with lung cancer in November 2019 revealed by hemoptysis. The clinical examination revealed a body mass index at 25 kg/m^2^, a preserved general well being (OMS score = 0) and bilateral supraclavicular lymphadenopathies. Abdominal exam was normal besides a midline laparotomy scar. Laboratory tests showed normal renal and hepatic functions. Complete blood count was normal (hemoglobin = 13 g/dl, white blood cells = 9700/l and platelets count = 193,000/l). The chest x-ray revealed spiculated opacity in the right lung (Figure 1).

**Figure 2.**
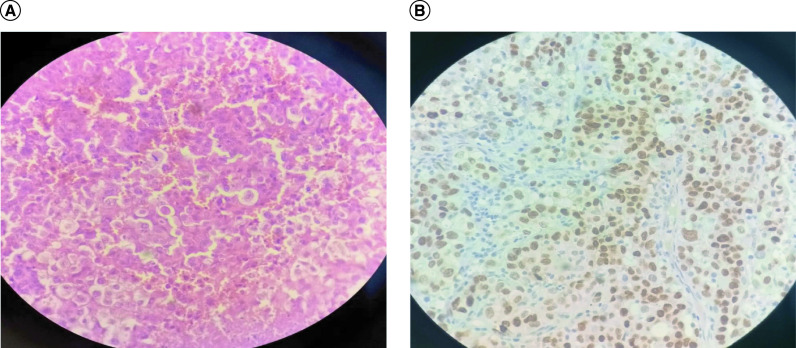
Histological examination of lymphadenopathy. **(A)** Hematoxylin and eosin staining showing tumoral proliferation. **(B)** Immunohistochemistry showing positive staining for TTF-1.

**Figure 3.**
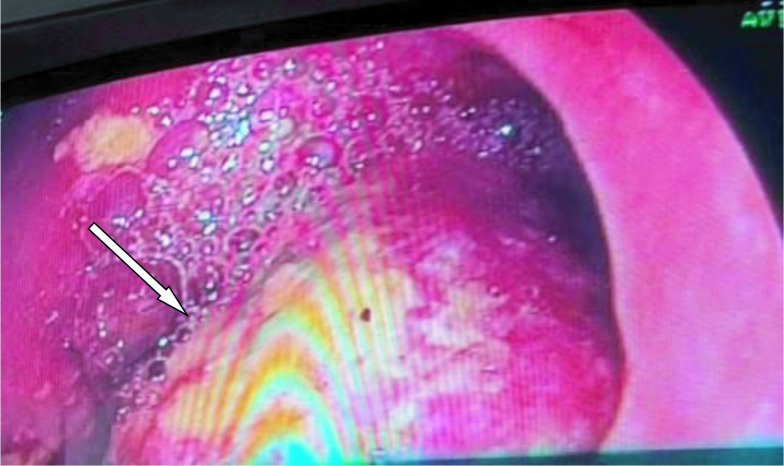
Ulcerated tumor in the pyloric antrum of the stomach.

**Figure 4.**
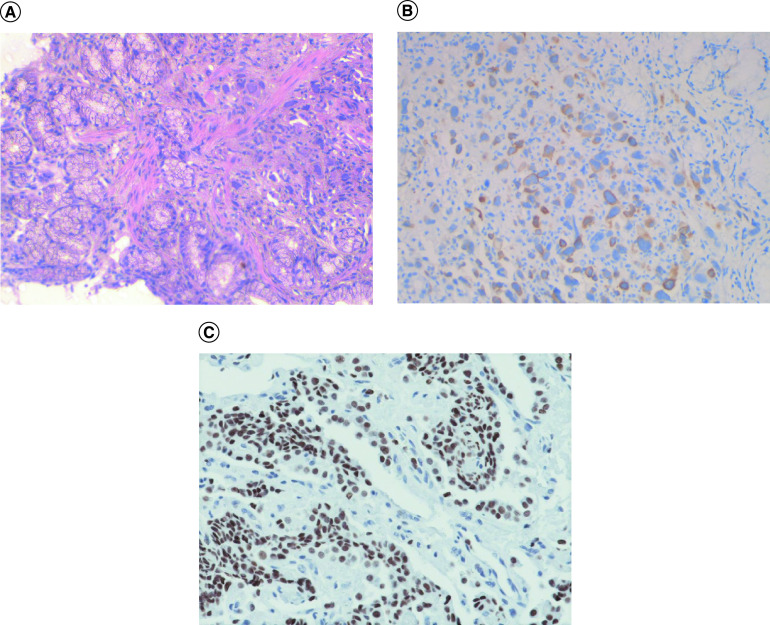
Histological examination of gastric metastasis. **(A)** Hematoxylin and eosin staining showing tumoral proliferation with rare glandular cavities. **(B)** Immunohistochemistry showing positive staining for CK7+. **(C)** Immunohistochemistry showing positive staining for TTF-1 (400×).

CT scan showed a 45-mm distal pulmonary mass with lymphatic extension. It also revealed a left sub diaphragmatic peritoneal node consistent with peritoneal carcinosis. There was no gastric parietal abnormality. Bronchial endoscopy confirmed the presence of the tumor. Histological examination of lymphadenopathy concluded to a poorly differentiated adenocarcinoma with bronchopulmonary origin (Figure 2). The tumor was staged T4N3M1, stage IV. Palliative chemotherapy based on gemcitabine-carboplatin was initiated. About 1 month later, the patient presented with melena resulting in severe anemia (hemoglobin level was at 5 g/dl). Upper endoscopy revealed an ulcerated tumor on the pyloric antrum obturating partially the lumen (Figure 3). Histological examination revealed that the cells expressed largely thyroid transcriptional factor-1 (TTF-1) and that the tumor was CK7+/CK20-. This pattern confirmed the diagnosis of gastric metastasis of an undifferentiated pulmonary adenocarcinoma (Figure 4). The patient passed away 1 month later.

## Discussion

Lung cancer is the most common cancer among men and the most common cause of cancer-related death in both men and women [1]. Half of the pulmonary cancers have already metastasized at the time of diagnosis [1]. The usual sites of metastases from lung cancer are lymph nodes, liver, bones, brain, adrenals and heart [2]. Rarely, it can metastasize to the GI tract and more unlikely to the stomach [3]. Gastric metastasis are mostly described in case of breast cancer (33%), lung (25%), melanoma (22%), head and neck cancers (6%) [3,4]. Gastric metastasis from lung cancer seems to be more frequent in smoking men aged over than 45 years old. The mechanism invoked in this atypical site of metastasis is that tumoral cells reach the stomach via swallowing sputum rich in cancer cells, which occurs more likely in smokers who are more susceptible to gastric mucosal damage than non smokers [5]. Only 2% of all gastrointestinal metastases are symptomatic [2]. They may be revealed by epigastric pain, hemorrhage, nausea, vomiting and anemia [1]. It may even result in gastric perforation or pyloric obturation [2,6]. Our patient presented with melena. In upper endoscopy, it appears usually as a submucosal tumor which may explain the high prevalence of asymptomatic tumors. Otherwise, it appears as bull’s eye sign, a ‘volcano-like’ or an umbilicated lesion usually located in the fundus or the cardia [6,7]. An infiltrating ‘linitis plastica’ pattern is largely less seen in case of lung cancer than in breast cancer [7,8]. In our case, the tumor was in the pyloric antrum. The diagnosis confirmation is based on pathology. The main difficulty is to differentiate between primary gastric cancer and gastric metastasis in the particular case of adenocarcinoma. In these cases, immunohistochemistry is compulsory to define the primary site even when the tumor is undifferentiated. In fact, TTF-1 is useful in the diagnosis of primary lung cancer. It is positive in the majority of lung adenocarcinomas and thyroid cancer while it is negative in gastric cancer, breast cancer and colorectal cancer [2,9]. Therefore, TTF-1 is recognized as a specific marker for primary lung cancer. Moreover, the CK7+/CK20- pattern is typical in lung cancer with a specificity of 90–100% while it does not exceed 40% in gastric cancer [2]. In our case, immunohistochemical staining was positive for TTF-1 and CK7. It was negative for CK20 which allowed confirming the diagnosis of gastric metastasis of a pulmonary adenocarcinoma. Management of symptomatic gastric metastases is controversial. Supportive treatment without surgery seems to provide longer survival. In the case of complicated metastasis such as hemorrhage, obstruction or perforation, the surgery remains justified to improve life quality [7]. Our patient had peritoneal carcinosis besides the gastric metastasis and the symptoms were contained with medical treatment thus we pursued palliative measures. As for prognosis, stage IV lung cancer is known to have poor prognosis with an approximate survival of 10–12 months under palliative chemotherapy. In the case of gastric metastases, the prognosis is even poorer with a survival ranging from 3 to 12 months [10]. For our patient, the survival did not exceed 1 month after gastric metastasis diagnosis.

## Limitations

The limitation of our study is that neither genetic study of the primary tumor nor the gastric metastasis could be performed since the financial conditions of the patient did not allow it.

## Conclusion

Gastric metastases of primary lung cancer constitute a rare phenomenon that every clinician must keep in mind. The main differential diagnosis is primary gastric cancer. Thus we highlight the importance of immunohistochemical staining for TTF-1 and for the pattern CK7/CK20 to confirm diagnosis.

**Executive summary**

- Gastric metastases from lung cancer are uncommon.
- The diagnosis is challenging since the symptoms are undistinguishable between primary and metastatic gastric cancer.
- Diagnosis is based on immunochemistry in particular the CK7 CK20 profile and thyroid transcriptional factor-1 staining.
- Prognosis is poor with an overall survival less than 12 months.
